# Use of a Laparoscopic Witzel Gastrostomy Without Gastropexy in Bariatric and General Surgery

**DOI:** 10.1007/s11695-020-04871-z

**Published:** 2020-08-25

**Authors:** Joshua Davies, Luise Pernar, Danielle Eble, Adolfo Z. Fernandez, Brian Carmine, Donald Hess, Cullen Carter

**Affiliations:** 1 Department of Surgery, Boston Medical Center, 1 Boston Medical Center Place, Collamore Suite D501, Boston, MA 02118 USA; 2 Department of Surgery, University of Washington, 329 Ninth Ave, Box 359796, Room 7EH-70, Seattle, WA 98104-9796 USA; 3 Department of Surgery, Wake Forest University Baptist Medical Center, Medical Center Boulevard, 5th Floor, Winston-Salem, NC 27157 USA

**Keywords:** Laparoscopy, Gastrostomy, Gastric bypass, Enteral access

## Abstract

**Background:**

Gastrostomy placement is the preferred means of long-term enteral feeding for patients who cannot eat by mouth. During laparoscopic gastrostomy, it is standard to perform gastropexy, apposing visceral and parietal peritoneum. In some settings, due to altered anatomy from prior surgery, direct apposition of the stomach to the abdominal wall is not possible. This study reports a series of cases where laparoscopic gastrostomy was performed via a Witzel approach without gastropexy.

**Methods:**

A retrospective chart review was performed of all patients at a tertiary academic medical center who underwent Witzel gastrostomy without gastropexy over a 3-year period. In each case, an 18-French tube was placed into the fundus of the stomach and secured with a purse-string suture. A 5-cm serosalized Witzel tunnel was created around the tube using running silk suture. No gastropexy was performed.

**Results:**

Six patients underwent 7 Witzel gastrostomy procedures. In three cases, patients had undergone prior major upper abdominal surgery where adhesive disease prevented gastropexy. In the other four cases, the patients had undergone prior gastric bypass with antecolic antegastric position of the roux limb. No patient suffered leak of gastric contents into the peritoneum, and there were no postoperative complications or mortality related to the gastrostomy.

**Conclusion:**

In cases where enteral access is necessary, and where the stomach cannot reach the anterior abdominal wall for gastropexy due to prior surgeries, a Witzel gastrostomy without gastropexy is a safe option which resulted in no morbidity or mortality in our series.

## Introduction

Enteral nutritional access is the preferred means of delivering nutrition to patients who require prolonged supplementation. For most patients, percutaneous endoscopic gastrostomy (PEG) can be safely performed with low complication rates and has thus replaced open gastrostomy as the procedure of choice in establishing gastric access [1, 2]. Image-guided percutaneous gastrostomy is also a safe means of gastric access and has outcomes similar to those of PEG [3]. While prior abdominal surgery was initially considered a contraindication to PEG, several authors have reported PEG to be a safe procedure even in those who have undergone major abdominal surgery [4, 5].

In some patients, such as those who have undergone upper abdominal surgery or gastric surgery, including gastric bypass, anatomical limitations or significant adhesive disease prevent safe percutaneous access to the stomach. In these cases, laparoscopic gastrostomy placement can allow safe access to the stomach and still offer the benefits of a minimally invasive technique [6]. However, a tenet of laparoscopic gastrostomy is fixation of the stomach to the abdominal wall (gastropexy) [7]. In some cases, due to either extensive adhesive disease or the presence of an antecolic antegastric roux limb, the stomach or remnant stomach will not reach the abdominal wall, thus precluding laparoscopic, open, or percutaneous gastrostomy. In such patients, a jejunal feeding tube may be placed in lieu of a gastric feeding tube. However, it is well established that jejunal feeding tubes are associated with higher complication rates and are inconvenient for patients due to frequent obstruction and the need for continuous rather than bolus feeding [8].

The aim of the current study is to describe a novel technique of laparoscopic “Witzel” gastrostomy without gastropexy as a means of gastrostomy placement when the stomach cannot reach the abdominal wall.

## Material and Methods

### Patients

A retrospective chart review was performed of all patients at a tertiary academic medical center who underwent “laparoscopic Witzel gastrostomy without gastropexy” over a 3-year period between 2015 and 2018. Data were collected on the age of the patient, the indication for enteral access, the reason safe gastropexy could not be performed, and the morbidity or mortality resulting from feeding tube placement. All patients received a silicone-based Halyard MIC® Gastrostomy Feeding Tube (Catalog no. 0112-18). Neither IRB approval nor patient consent was required for our retrospective review.

### Operative Procedure

Patients are positioned in the supine position with both arms extended. The peritoneum is accessed with a Veress needle and insufflated. A camera trocar is placed in the left mid abdomen, and two working trocars are placed in the right abdomen. An assistant trocar is placed in the left upper quadrant as necessary. Trocar placement varies based on the presence of intra-abdominal adhesions or the primary indication of the procedure, for example, if a perforated marginal ulcer was repaired.

**Fig. 1:**
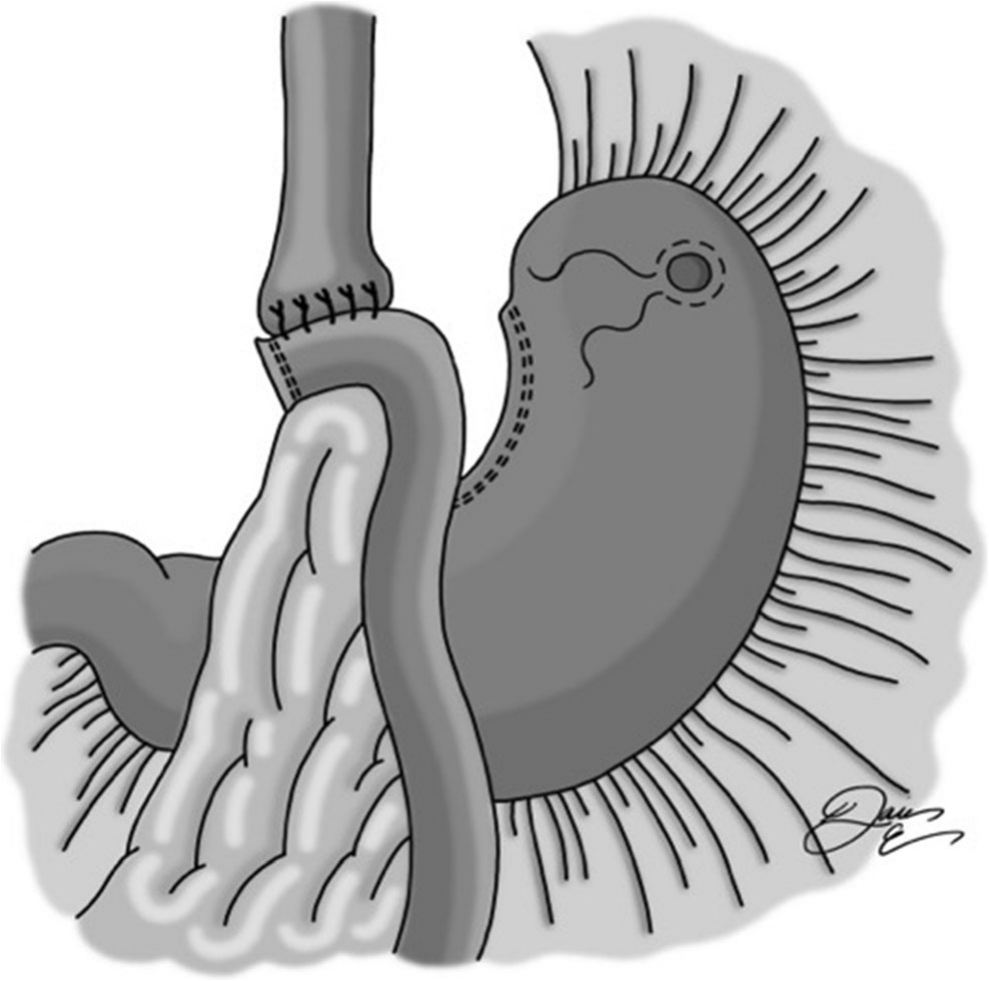
Gastrotomy made in fundus, followed by placement of purse-string silk suture. Gastrostomy inserted, and purse-string tied

**Fig. 2:**
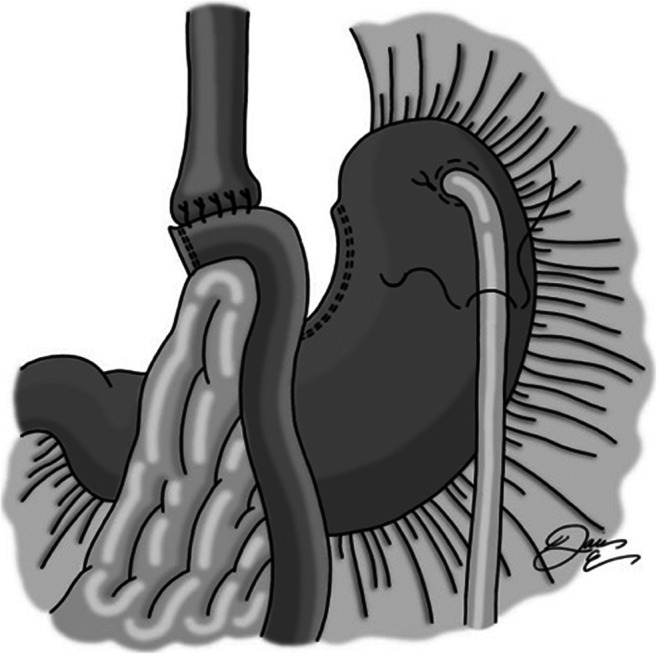
Second silk suture is placed and tied, approximately 5 cm distally, taking seromuscular bites of stomach on either side of the tube

**Fig. 3:**
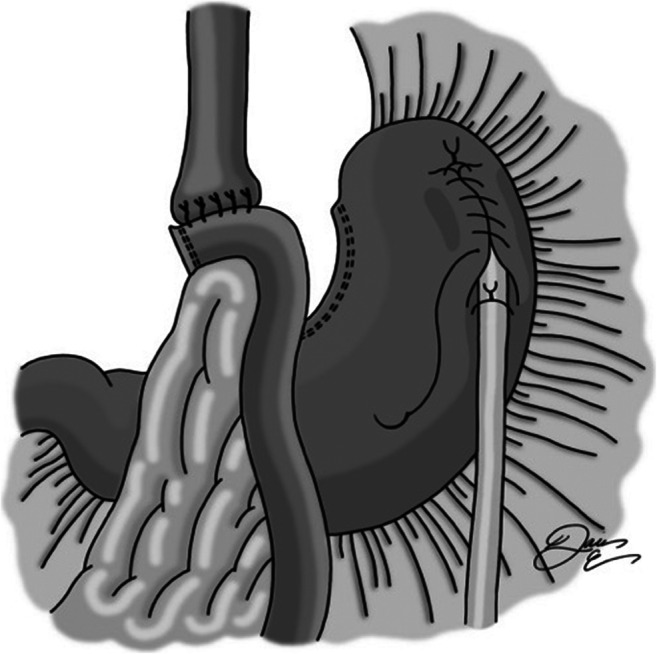
A proximal running silk suture is placed and ran distally toward the previously placed distal suture. Seromuscular bites of stomach taken on either side of the tube

**Fig. 4:**
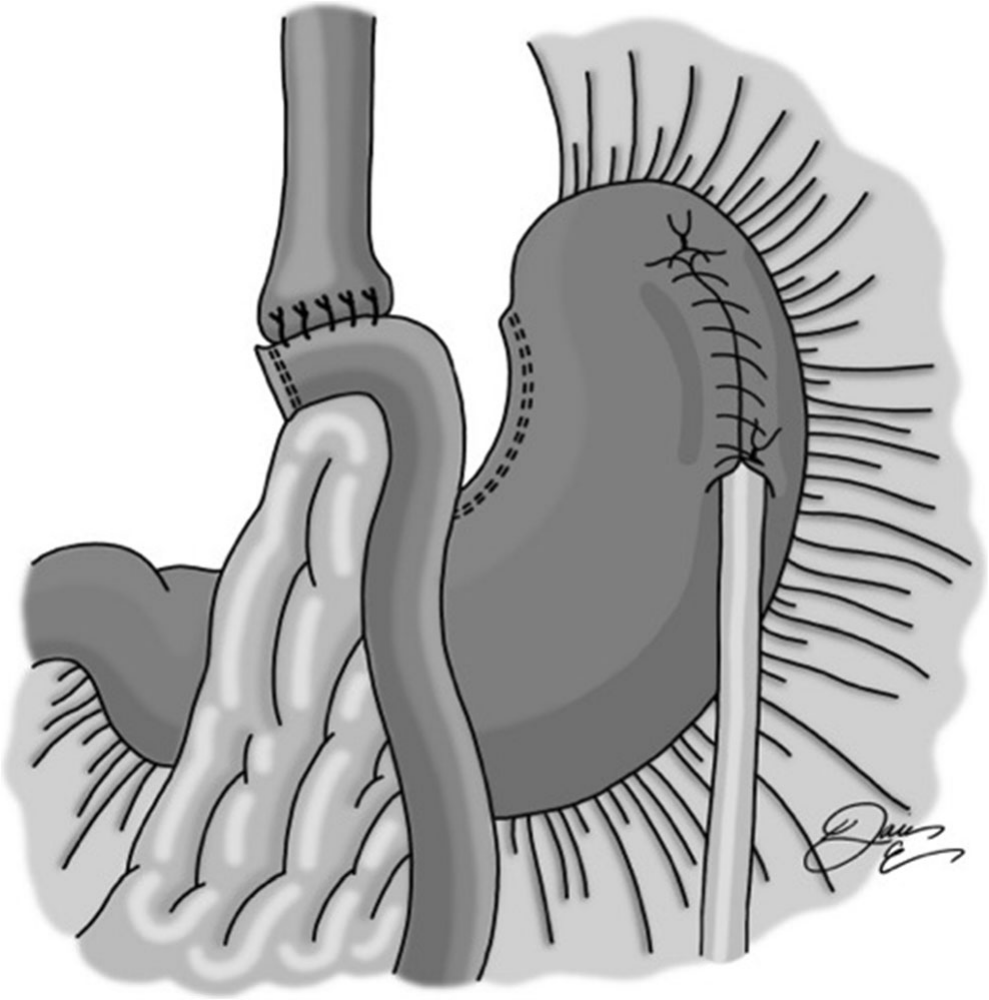
Tie running silk suture to distal silk suture, completing the Witzel tunnel

A location in the fundus of the stomach free of adhesive disease or extensive scarring is identified, and a gastrostomy is created with cautery and surrounded by a silk purse-string suture (Fig. 1). An 18-French balloon tip gastric feeding tube is placed into the stomach and then secured with the purse-string suture. The balloon is then inflated. A second suture is placed approximately 5 cm distally, taking seromuscular bites of stomach on either side of the tube (Fig. 2). This is tied snugly so as to wrap serosa around the feeding tube. The suture is left long so that the next suture can be secured to it. Next, a third silk suture is used to create a serosal tunnel, starting proximally over the gastrostomy site, and then continued in a running fashion with seromuscular bites of tissue on either side of the tube moving gradually toward the previously placed distal suture (Fig. 3). This suture is then tied to the distally placed suture, creating a seromuscular Witzel tunnel measuring approximately 5 cm in length (Fig. 4).

Not pictured is the method used to bring the feeding tube into the peritoneal cavity. Our preference is to place a 5-mm port at the desired location of tube entry. We then place a grasper through any right-sided port, into the peritoneum, and then out the 5-mm port placed at the tube entry site. The port is then removed, leaving only the grasper exiting the abdominal wall, and the tip of the feeding tube is carefully retracted into the peritoneum. A “peel-away” sheath could also be used to introduce the tube if desired. Finally, we routinely secure the tube to the skin using two nylon sutures to prevent accidental dislodgement.

## Results

Between September, 2015 and August, 2018, 6 patients underwent 7 Witzel gastrostomy procedures. Details of each patient are presented in Table 1. Three patients had undergone prior major non-bariatric upper abdominal surgery. In one of these patients, it was known that an antrectomy had been performed. The other two patients had upper midline incisions but did not know exactly what surgery had been performed previously. The indication for gastrostomy was dysphagia due to head and neck cancer in two cases and critical illness resulting from myasthenia gravis in the third. In all three of these cases, severe adhesive disease prohibited safe gastropexy, and thus, the Witzel technique without gastropexy was used, placing the feeding tube into the more proximal part of the stomach.

**Table 1:** Patient characterisitics and operative details

Patient	Age	BMI	Feeding tube indication	Reason for Witzel	Operative time	EBL	Leak from gastrostomy	Complications/mortality related to gastrostomy
1	61 F	37	Perforated marginal ulcer	Antecolic roux limb, need for enteral access	3 h 2 m	40 cc	No	None
2	76 F	23.5	Dysphagia secondary to metastatic anaplastic thyroid carcinoma	Severe adhesions from prior operations	2 h 42 m	50 cc	No	None
3*	36 F	36.5	Intolerance of oral feeds	Antecolic roux limb	2 h 30 m	10 cc	No	None
3*	37 F	25.2	Marginal ulcer and malnutrition	Antecolic roux limb	1 h 57 m	10 cc	No	None
4	86 M	27.9	Myasthenia gravis	Dense adhesions, two prior perforated peptic ulcers, open cholecystectomy	3 h 12 m	20 cc	No	None
5	49 F	27.9	Chronic nausea, malnutrition	Antecolic, antegastric Roux limb	2 h 4 m	40 cc	No	None
6	57 F	18.5	Dysphagia from invasive SCC	Prior Antrectomy/Billroth II for peptic disease; severe adhesive disease	3 h 26 m	40 cc	No	None

*Same patient

In the remaining four cases, the patients had undergone prior gastric bypass with antecolic antegastric position of the roux limb. The indication for feeding tube placement in these cases was severe malnutrition in three cases and perforated marginal ulcer in one case. In each case, the roux limb and its mesentery was densely adherent to the antrum and body of the stomach, and thus prevented safe gastropexy. Again, a Witzel technique without gastropexy was performed, placing the tube into the fundus in each case.

It was not routine in any of these cases to obtain a routine postoperative radiologic study to assess placement of the feeding tube or subclinical leak. We did obtain a fluoroscopic contrast tube study on one of the head and neck cancer patients 1 month after tube placement after reported copious drainage around, though this did not show displaced gastrostomy or leak. No patient suffered leak of gastric contents into the peritoneal cavity, and no drains were placed. There were no postoperative complications related to the Witzel gastrostomy procedure.

In five out of six patients, the feeding tube was subsequently removed at an interval of at least 2 months after placement, with the maximum of 6 months after placement. There was no added difficulty in the office-based removal of these tubes. One of the patients with a head and neck cancer died from complications secondary to their malignancy 2-month status postgastrostomy tube placement; thus, this tube was never removed. There was no intraperitoneal leak of gastric contents in any patient, and the skin healed quickly in each case.

## Discussion

We hereby present a series of cases where gastric access was performed when the stomach could not reach the abdominal wall for gastropexy. In each case, a novel technique of laparoscopic Witzel gastrostomy without gastropexy was performed.

The concept of creating a serosal tunnel around a gastric feeding tube was first described by Witzel in 1891 [9]. It is of interest that Witzel’s initial description involved placement of a feeding tube into the stomach, and not the jejunum, as is often thought. In 1894, Caird published the first English-language description of the same technique in two patients who suffered esophageal stricture [10]. He cited major benefits of the technique to be minimization of leakage of gastric contents to the skin, as well as rapid closure of the fistulous tract should the tube become unnecessary. Of note, in Caird’s description, gastropexy was performed.

In 1982, Johnson et al. described the first series of patients who underwent placement of a Witzel gastrostomy without any type of gastropexy [11]. In their series of 361 cases, minimal morbidity was performed, and only twice was reoperation indicated for a complication related to gastrostomy. The authors found that this technique allowed for faster placement of the tube and felt that allowing the stomach to remain in anatomic position was an advantage. Of note, the tubes placed in this series were primarily placed for postoperative gastric decompression. Of interest, tubes were removed at an average of 14 days after placement, and there was no occurrence of intraperitoneal leakage of gastric contents.

In 2006, Hsieh et al. made the first report of laparoscopic Witzel gastrostomy [12]. They report using the technique in 42 patients who required gastric access in the setting of advanced head and neck cancer. They reported no major complications, and the rate of minor complications such as superficial wound infection, balloon rupture, or chronic granulation was 11%. Of note, gastropexy with three sutures was performed to minimize the risk of peritoneal leakage of gastric contents in each case. The authors felt the major benefit of creating a Witzel tunnel was to minimize reflux of gastric contents onto the skin.

The present study is the first description of performing gastrostomy without gastropexy using a laparoscopic Witzel technique. We believe the technique described is very useful in bariatric surgery and in general surgery cases where gastric access is required but the stomach cannot reach the abdominal wall to perform gastropexy. We believe this technique is a safe alternative to jejunostomy placement and can avoid complications inherent to jejunostomy.

As noted, in our series, patients experienced no peritoneal leak of gastric contents upon removal of the feeding tube. We believe that a fibrinous tract likely forms around the exposed intraperitoneal portion of the tube akin to the tract known to form around a “T-tube” placed during common bile duct exploration. We believe this tract prevents intraperitoneal leakage upon tube removal. The technique we describe here is applicable to all patients in which gastropexy is not possible and did not differ among those with variable body habitus (BMI ranged from 18.5 to 37).

Limitations of the current study include the limitations inherent to a retrospective study, as well as the small number of patients included. In addition, we are unable to comment on the ability to change a gastrostomy feeding tube at the bedside as we have not personally attempted this. Thus, the technique may not be ideal in circumstances where a permanent feeding tube is desired. If a tube placed in this fashion does need to be changed, we recommend changing the tube over a wire under fluoroscopic guidance so the tract is not lost. Finally, no patient in this series experienced accidental tube dislodgement. We feel that accidental dislodgement could result in intraperitoneal leak of gastric contents, and we do recommend using extra care in securing the tube to the skin to prevent this complication.
